# Complete genome sequence of *Salmonella enterica* bacteriophage SeKF_80, isolated from wastewater in British Columbia

**DOI:** 10.1128/mra.01031-24

**Published:** 2024-12-19

**Authors:** Thomas Guy, Colleen Harlton, Siyun Wang, Karen Fong

**Affiliations:** 1 Summerland Research and Development Center, Agriculture and Agri-Food Canada, Summerland, British Columbia, Canada; 2 Faculty of Land and Food Systems, The University of British Columbia, Vancouver, British Columbia, Canada; Queens College Department of Biology, Queens, New York, USA

**Keywords:** bacteriophage, *Salmonella*, microbiology, food microbiology, genomics

## Abstract

*Salmonella enterica* is a Gram-negative inhabitant of the gastrointestinal tract of warm-blooded animals and commonly implicated in foodborne illness. Here, we describe the isolation of *Salmonella enterica* phage SeKF_80. The 89,965 bp genome contains 174 predicted coding sequences with 44 predicted functions. Phage SeKF_80 shares species-level similarity with *Salmonella* phages 7–11.

## ANNOUNCEMENT

*Salmonella enterica* is a foodborne pathogen causing a significant disease burden globally (1). Here, we present the genome of SeKF_80, a lytic Podophage with an elongated capsid, which was found to infect via the outer membrane protein TolC of *Salmonella enterica* serovar Typhimurium strain CDC 6516–60 (ATCC 14028; CP001363), a strain originally isolated from chickens. This phage was selected for sequencing due to the importance of its binding receptor, TolC, in both virulence and antimicrobial resistance (2).

**Fig 1:**
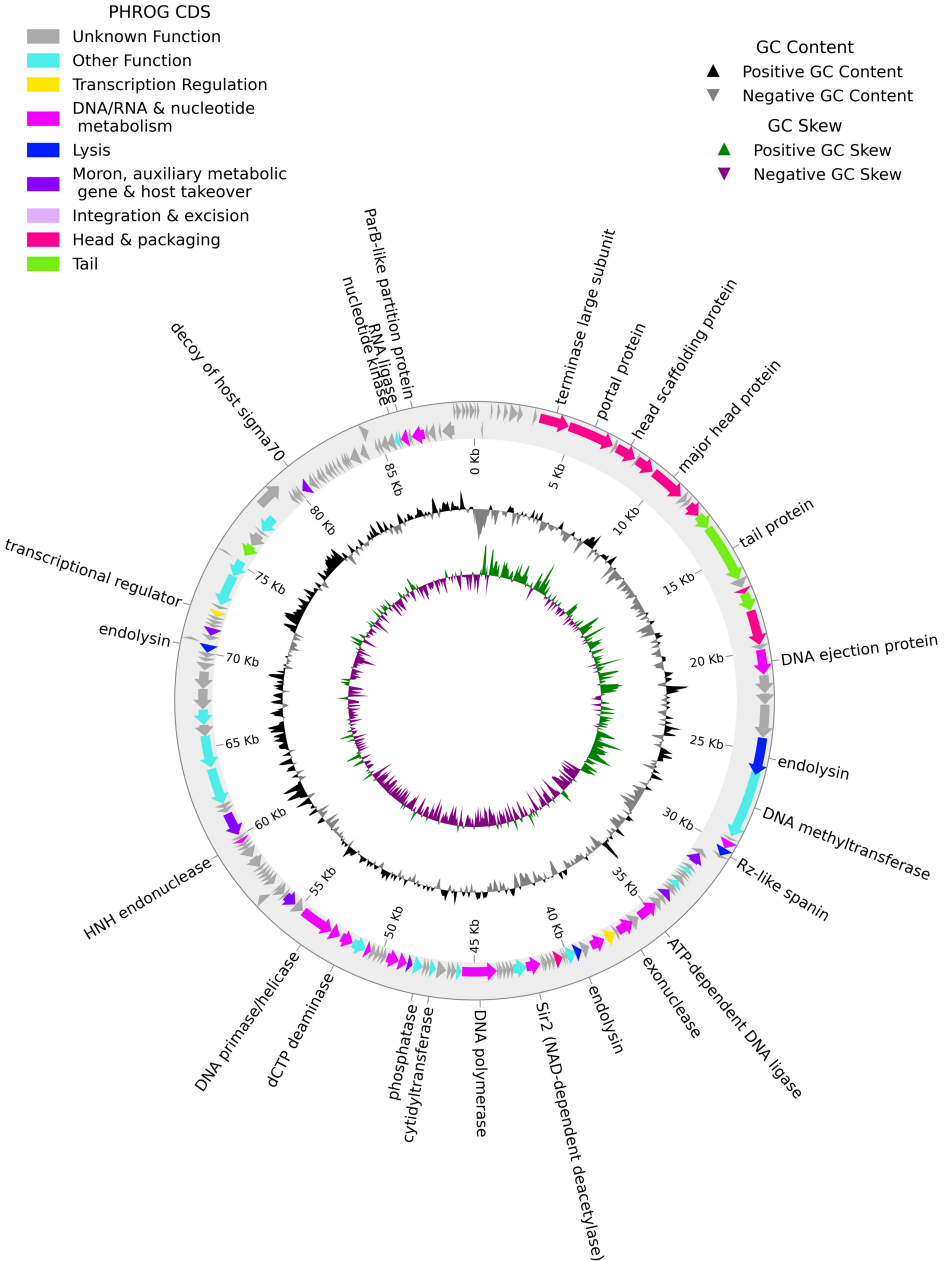
Whole genome plot of *Salmonella enterica* phage SeKF_80.

Phage SeKF_80 was isolated through the enrichment of wastewater from Lake Country, British Columbia, Canada, 50°01’30”N 119°23’10”, followed by filtration through a 0.22 µm nylon filter (VWR; Radnor, PA, USA). The phage was plaque purified over five rounds on *Salmonella* Typhimurium ATCC 14028 at 37°C on tryptic soy agar (BD; Sparks, MD, USA) with the double agar overlay method (3). Phage genomic DNA was isolated and purified with the phenol-chloroform method (4) and prepared as Illumina NxSeq AmpFREE low-DNA libraries. DNA was sequenced in paired-end 250 bp reads using v2 500-cycle chemistry on an Illumina MiSeq platform, software version 4.0.0.1769. The 58,444 raw reads were trimmed with fastp v0.23.2 (5), normalized with bbnorm, and subsampled with Seqtk v1.4 using a coverage cutoff of 70. The reads were assembled with SPAdes v3.15.5 (6). Annotation of the genome assembly was completed with Pharokka v1.5.0 (7). Coding sequences (CDS) were predicted using PHANOTATE (8), tRNAs were predicted with tRNAscan-SE v2.0 (9), tmRNAs were predicted with Aragorn (10), and CRISPRs were predicted with CRT (11). Assignment of functional categories was accomplished by linking each CDS to the PHROGs (12), VFDB (13), and CARD (14) databases using MMseqs2 (15) and PyHMMER (16). Contigs were matched to their closest hit in the INPHARED database (17) using mash (18). The output of Pharokka was used to run Phold v0.2.0, providing additional annotations via protein structural homology (19). Phold was also used to generate the genome plot (Fig. 1). The quality control of raw reads, assembly, and annotation was completed on Ubuntu version 20.04.6. The nearest relatives were found using megaBLASTn v2.16.0 (20), followed by VIRIDIC to compare intergenomic similarities (21). Finally, PhageTerm v1.0.12 was used to predict the genome packaging on the Galaxy Pasteur web server (22, 23). Default parameters were used, except where otherwise noted.

Assembly resulted in a single contig 89,965 bp in length, with 137-fold coverage. The genome has a mean G + C content of 44.2%, with 174 predicted CDS, 56 of which have predicted functions, including seven tRNA genes. Analysis of the genome packaging via PhageTerm did not predict a packaging class but suggested that SeKF_80 has redundant ends, two preferred termini on the forward strand, and several possible termini on the reverse strand. A megaBLAST search against NCBI’s nucleotide collection revealed the closest relatives to be *Salmonella* phages 7–11 (NC_015938) and SE131 (NC_070974). Further analysis using VIRIDIC confirmed species-level similarity between SeKF_80 and 7–11 (95.8%) and genus-level similarity between SeKF_80 and SE131 (92.8%) (21). SeKF_80 belongs to the genus *Moazamivirus* and the family *Grimontviridae*.

## Data Availability

The genome sequence was deposited under GenBank accession number OR863363. The version described in this paper is the second version. The raw sequencing reads are available under SRA BioProject PRJNA1166150.
